# Supplementary material for: COVID-19 mitigation measures in primary schools and association with infection and school staff wellbeing: An observational survey linked with routine data in Wales, UK
Source: PLoS One. 2022 Feb 28;17(2):e0264023. doi: 10.1371/journal.pone.0264023 (PMC8884508; doi:10.1371/journal.pone.0264023)
Supplement: Attachment — Submitted filename: Response to reviewers.docx [file pone.0264023.s007.docx]

**Reviewer #1:**

**The questions was phrased with multiple alternatives (see below) whereas the analyses are done separately as dichotomous exposures. It is not clear whether the research question is relative or related to the exposure as whole. I would have expected that Keeping 2 metres from STAFF was treated as one variable (exposure as whole), and that this variable should be tested for significance before interpreting the significance of the subvariables (rarely, some of the time, most of the time, always) I think authors need to clarify this.

This was not related to ordinal data, but to the validity of the analysis. Before doing pairwise comparisons of separate values within a variable (i.e. testing “sometimes” versus “never/rarely”) the variable “keeping 2 meters distance” should be tested for significance. The p-value for the whole variable can be calculated in two ways:
1. A likelihood ratio test comparing a model containing the variable vs a model not containing the variable
2. A wald test that tests the joint significance of beta1 = beta2 = 0 (where beta1 is the coefficient for sometimes and beta2 is the coefficient for most of the time/always)

I believe pairwise comparisons (i.e. testing “sometimes” versus “never/rarely”) should be limited to significant variables to make sense, and the the p-value for the whole variable should be presented in the table alongside the pairwise comparisons.

I have no further comments**

On my behalf of the co-authors, we would like to thank you for taking the time to read our revised manuscript. We are glad that you are satisfied that we have addressed the useful comments provided and that this has improved the flow and readability of the paper.

Thank you for drawing further attention and providing additional clarity regarding your previous comment about the multivariable analysis. Further to the changes in revision one, we have addressed this point as you have suggested by performing a likelihood ratio test for the variable *keep two metres* as a whole variable i.e. survey response categories. We have assessed the goodness of fit of two alternative models including and excluding this whole variable. The output from this test shows a significantly improved difference with the inclusion of the keep two metres from staff variable (p=0.03), a variable that we found to be associated with increased likelihood of COVID-19 positive test in the school. For keep two metres from pupils, the output presents a likelihood ratio of p=0.1 (borderline significant at the 10% level), though we did not report on this variable as we found no association with outcomes in multivariable models. To ease interpretation of Odds Ratios, we created ordinal categories relating to level of exposure.

We have addressed this within the manuscript, including stating within the methods, results and in the Table 3 footnote. Please find below further information of these revisions:

***Quantitative analysis, page 8-9, line 191-198.***

*Items with multiple category responses or continuous numerical values were assigned ordinal categories to ease interpretation. For example survey response categories for keep two metres from pupils/staff included i) never, ii) rarely, iii) some of the time, iv) most of the time, v) always, with combined ordinal categories for analyses of i) never/rarely, ii) some of the time, iii) most of the time/always. For these variables, likelihood-ratio tests of variables as whole were performed to assess goodness of fit between models including and excluding variables for the primary outcome.*

***Table 3, page 16, line 282-287.***

*Univariable (model 1) and multivariable (model 2) logistic regression models of self-reported school-based mitigation measures (survey) and school-level probability of any positive COVID-19 case in school (SAIL). OR: Odds Ratio; 95% CI: 95% confidence intervals; p<0.05**, p<0.1*; adjusted for school size, proportion of pupils eligible for free school meals, clustered by school. Model 2 likelihood-ratio test keep two metres from pupils (p=0.1) and staff (p=0.03). See S3 Appendix for variable codebook.*

Finally, we have replaced *2-metre* to *two metre* throughout the manuscript, and have updated table layouts.
